# The study on the effect of flotation purification on the performance of α-hemihydrate gypsum prepared from phosphogypsum

**DOI:** 10.1038/s41598-021-04122-w

**Published:** 2022-01-07

**Authors:** Mingxia Du, Jinming Wang, Faqin Dong, Zhaojia Wang, Feihua Yang, Hongbin Tan, Kaibin Fu, Weiqing Wang

**Affiliations:** 1 School of Environment and Resource, Southwest University of Science and Technology, Mianyang, 621010 Sichuan China; 2 State Key Laboratory of Mineral Processing, Beijing, 100160 Beijing China; 3 Key Laboratory of Solid Waste Treatment and Resource Recycle Ministry of Education, Southwest University of Science and Technology, Mianyang, 621010 Sichuan China; 4 Beijing Building Materials Academy of Sciences Research Co., Ltd., Beijing, 100041 China; 5 State Key Laboratory of Solid Waste Reuse for Building Materials, Beijing, 100041 China

**Keywords:** Mineralogy, Pollution remediation

## Abstract

Phosphogypsum (PG) is a massive industrial solid waste. In this paper, PG was purified by flotation method, and α-hemihydrate gypsum (α-HH) was prepared by the autoclaving method. The morphology of α-HH was adjusted by adding different doses of Maleic acid and Aluminium sulfate. The results showed that after flotation purification, the impurity content in PG was significantly reduced, the soluble phosphorus content decreased from 0.48 to 0.07%, the PG purity increased from 73.12 to 94.37%, and the PG whiteness risen from 19.4 to 40.5. Then the performance of α-HH prepared from PG before and after purification was compared. Fixing the amount of aluminium sulfate at 0.2 wt%, the reaction temperature at 140 °C, and the reaction time at 120 min, the average length/diameter ratio of α-HH crystals decreased from 7.2 to 0.6 as the amount of Maleic acid increased from 0 to 0.17 wt%. When the amount of Maleic acid was 0.13 wt%, the α-hemihydrate gypsum reached the best mechanical properties. The mechanical strength of high strength gypsum prepared from PG concentrate was significantly better than that of raw PG, indicating that flotation purification can effectively improve the performance of PG. In this study, a new method of PG purification and resource utilization was proposed.

## Introduction

Phosphogypsum (PG) is a massive industrial solid waste generated by the wet process of phosphoric acid preparation, which typically produces 5 tons of PG for every 1 ton of phosphoric acid produced. The cumulative global emissions of PG are estimated to be about 6 billion tons and are increasing at a rate of 150 million tons/year. It is estimated that the total amount of PG stockpiles will grow to twice the current level by 2025 to 2045^1–3^. The main component of PG is CaSO_4_·2H_2_O, which also contains a variety of impurities, such as SiO_2_, soluble phosphorus, fluorine, organic matter, and some containing heavy metal ions and radioactive elements. The disadvantages of PG such as poor water resistance and uneven particle size distribution lead to its inferiority to natural gypsum in terms of usability, hardness, and whiteness, thus limiting its application areas and making it challenging to be resourcefully utilized^4–6^. Currently, only 15% of PG is utilized, while the remaining portion can only be stockpiled in large quantities^7^. The piled PG occupies a large amount of land resources and the long time piling will lead to dust flying to pollute the air and the leaching of soluble phosphorus and fluorine to pollute the soil and water bodies^8,9^. Therefore, it is of environmental importance and urgency to choose a reasonable method to purify and resourcefully utilize PG.

The currently commonly used PG pretreatment methods can partially solve the impurity problem, such as using high-temperature calcination to obtain hemihydrate or anhydrous gypsum for use as cement and slag composite cementitious materials^10,11^; using calcium hydroxide to neutralize the acid in PG^12^; reducing the retarding time of PG by ammonia pretreatment^13^; and using citric acid solution pretreatment to convert phosphorus and fluorine in PG into citrate that can be removed by water washing^14^. However, all these methods may increase the treatment cost and cause secondary pollution. Moreover, due to the differences and complex composition of phosphate ores in different regions, it is difficult to remove harmful impurities from PG by a single method effectively. As an efficient and environmentally friendly method, flotation is now widely used to separate and purify pyrite, chalcopyrite, sulfide ore, rare earth ore and other minerals^15–18^. However, there were less studies on the use of flotation process for phosphogypsum purification, and the current relevant studies include the purification of phosphogypsum by flotation method by Wang et al., after purification, the whiteness of phosphogypsum was significantly increased, the purity reached 96.5%, and the soluble phosphorus and fluorine content were lowered to the national standard^19^; Dai et al. used a combination of flotation chemical methods to remove quartz from phosphogypsum by flotation, and then obtained gypsum powder with a whiteness of 95% and a purity of 93% by acid leaching and calcination^20^; For the treatment of high-silica phosphogypsum, Jiang W et al. used a combined process of flotation desliming and desilication, and the results showed 99% impurity removal from phosphogypsum, gypsum purity close to 99%, and gypsum productivity of 80%^21^. For the consideration of treatment cost, process complexity, treatment effect and differences in phosphogypsum characteristics, flotation was proposed as a method for the purification of PG.

At this stage, much research has been done on the resource utilization of PG. For example, PG is used as construction materials such as hard gypsum board^22^, foam concrete^23^, calcium sulfate whiskers^24^, no-burn bricks^25^, ceramics^26^, and lime-fly ash-phosphogypsum binder^27^. However, the amount of PG in the above-mentioned products is relatively small and cannot achieve the purpose of consuming it in large quantities. In addition, there are problems such as the relatively low added value of the product and the high cost of processing technology, which prevent it from being widely used. The α-hemihydrate gypsum (α-HH) has the advantages of high strength, lightweight, has no pollution, and good biocompatibility^28,29^. Currently, it has been widely used as a high value-added gelling material in the construction field^30^. The preparation of α -hemihydrate gypsum by phosphogypsum has attracted the attention of scientists and technicians. Mi et al. used the “semi-liquid method” to prepare α-HH from phosphogypsum, and the product obtained by mixing 0.1% Maleic acid at pH = 7 had the best morphology, with a 3d compressive strength of 43.7 MPa and a 3d flexural strength of 10.5 MPa^31^; Lu et al. used phosphogypsum as raw material and studied the effect of the number of cycles on crystal morphology and mechanical strength by using the atmospheric pressure CaCl_2_ solution method, and found that high strength gypsum with 95% purity could be prepared after 6 cycles^32^. In the preparation of α-HH from phosphogypsum, the hemihydrate gypsum usually has a long columnar shape and incomplete crystals due to its growth characteristics, so it is necessary to add a crystal transfer agent to assist crystal growth during the conversion process to obtain more dense and regular crystals. Yang et al. used Mg^2+^, Al^3+^, and Fe^2+^ ions as transcrystallizing agents to study their effects on α-HH crystallization, and the results showed that transcrystallizing agents accelerated the crystallization rate of gypsum crystals, increased the crystal surface energy, critical nucleation radius, and transformed α-HH crystals from long columnar to short columnar crystals^33^. Therefore, using PG as raw material and adding transcrystallizer to assist crystallization to prepare α-HH not only realizes resource recycling, but also protects natural gypsum resources and realizes sustainable development of environment, economy and society. Due to the preparation of α-HH by atmospheric salt solution method, the equipment needs to be in high salt medium for a long time, which is easy to cause serious corrosion of equipment and cannot be widely used in industry.

This study used a combination of ball milling and forward and reverse flotation to purify and purify phosphogypsum. The autoclaving method was selected to convert PG raw ore and concentrate into α-HH separately. The effects of different doses of maleic acid on the crystal morphology and properties of α-HH were discussed, and the optimal amount of transcrystallizing agent was obtained. The performance differences between the α-HH products prepared from PG before and after purification were compared. In this study, phosphogypsum was purified into higher purity calcium sulfate dihydrate and used in the construction field, which solved the problems of low utilization of phosphogypsum and low added value of the prepared products and reduced the mining of natural gypsum. The results of the study have important guiding significance for the resource utilization of phosphogypsum.

## Experimental method

### Materials

PG used in this study was obtained from a large phosphorus chemical company in Deyang, Sichuan, China. The samples were naturally dried at room temperature, mixed, shrunk, and weighed 200 g per bag for the test. Pine oils, Methyl isobutylcarbinol (MIBC), Tributyl phosphate (P86) and Dodecylamine were produced by Shanghai RHAWM Technology Development Co., Ltd., and Dodecyl trimethyl ammonium chloride (1231) was produced by Tianjin Kermel Chemical Reagent Co. The crystal modifier used in the test were Maleic acid and Aluminium sulfate, both produced by Chengdu Chron Chemical Co. All the reagents in the test were analytically pure. All reagents used in flotation which need to be diluted to a certain concentration by adding purified water, the resistivity of purified water is 18.25 MΩ.

### Flotation experiment

The flotation test first weighed 200 g of PG raw ore for ball milling (GSDM-003A fine grinding machine, Beijing GOSDEL POWDE & TECHNOLOGY Co., Ltd., China), with material to ball ratio of 3:1 and 500 ml of water, and the fineness of grinding was adjusted by different grinding times. The milled slurry was put into a 1L single tank flotation equipment (XFDIV, Jilin Province Prospecting Machinery Factory, China), stirred for 3 min, and reverse flotation was carried out by adding frothing agent and scraping the froth for 5 min to float out organic matter and microfine mud from phosphogypsum to obtain tailings1. Finally, gypsum concentrate was flotation by adding dodecylamine, and the product in the tank was tailings2. All test samples were dried at a temperature of 42 °C and used to test their yield, chemical composition, purity, whiteness, physical phase, morphology, etc. The productivity of concentrate and tailings is calculated according to formula (1), (2). The test water was laboratory tap water.

1 $${\text{Concentrate}}\;{\text{productivity}} = \left( {{\text{Concentrate}}\;{\text{quality/Raw}}\;{\text{ore}}\;{\text{quality}}} \right)*100\%$$

2 $${\text{Tailings}}\;{\text{productivity}} = \left( {{\text{Tailings}}\;{\text{quality/Raw}}\;{\text{ore}}\;{\text{quality}}} \right)*100\%$$

### Preparation of α-HH

The α-HH was prepared by autoclave method. The main steps included hydrothermal synthesis reaction, high-temperature drying, and characterization. The specific procedure was as follows: 2 g of PG concentrate (purified phosphogypsum by flotation), solid–liquid ratio 1:1^49^, was weighed and hydrothermally reacted in a 50 ml autoclave at 140 °C under fully closed conditions (the pressure of operation is 3 MPa), and the sample was quickly removed after 2 h of reaction, the upper liquid layer was poured out and dried at 120 °C for more than 12 h. The dried sample was stored in a desiccator and observed under a scanning electron microscope (TM-1000; HITACHI Co. Ltd., Tokyo, Japan) at a magnification of 1000 times to study whether there was any change in the crystal morphology. The effect of the amount of transcrystallizer on the crystal morphology of the prepared products was investigated. When the SEM image shows that it contains a large number of short columnar crystals, the reaction conditions required for the subsequent concentrate and raw ore comparison tests are reached, and the reaction is completed^34^. To further verify the differential changes of PG concentrate in the hydrothermal reaction with PG raw ore, PG raw ore was used as a control group to prepare α-HH under the same experimental conditions and compared with the characteristics of α-HH prepared from PG concentrate.

### Characterization

The chemical composition of PG was analyzed using a sequential wavelength dispersive X-ray fluorescence spectrometer (XRF; Axios advanced, PANalytical B.V, Netherlands) with a tungsten target, tube pressure 60 kV, tube current 10 mA. A scanning electron microscope (TM-1000; HITACHI Co. Ltd., Tokyo, Japan) was used to observe the PG crystal morphology. The L/D ratio of α-HH crystals was measured by Nano measurer. The thermal stability of PG was analyzed using a simultaneous thermal analyzer (TG-DSC; SDT Q600, TA Instruments, America) to characterize PG further and determine the water of crystallization content. Chemical functional groups were characterized using a Fourier transform infrared spectrometer (FTIR; Spectrum One, Perkin Elmer Corporation, America) with a scan range of 500–4000 cm^−1^. A whiteness tester (SBDY-1P, Shanghai Yuefeng Instrumentation Co. China) was used to determine the PG whiteness. The soluble phosphorus content in the samples was determined by the phosphorus-vanadium-molybdenum yellow double wavelength photometric method in the People's Republic of China Building Materials Industry Standard “Method for the determination of phosphorus and fluorine in phosphogypsum” (JC/T 2073)^35^. Determination of water content attached to PG, purity of calcium sulfate dihydrate in accordance with the Chemical Analysis Method of Gypsum (GB/T 5484-2012)^36^.

The water requirement of the standard consistency of the prepared products was determined with reference to the standard of “Determination of the physical properties of net gypsum slurry for construction” (GB/T 17669.4-1999)^37^. The setting time of the product was measured with reference to the standard of “Determination of physical properties of net gypsum slurry for construction” (GB/T 17669.4-1999)^37^. The flexural and compressive strengths of high strength gypsum in different periods (2 h, 3d) were prepared, maintained, and measured with reference to the standard “α-type high strength gypsum” (JC/T 2038-2010)^38^. Weigh the appropriate amount of α-bassanite powder and mix it according to the w/c of 0.4, pour it into three 40 mm × 40 mm × 160 mm test molds, shake and compact them, let them stand for 30 min, take off the molds, and cure them for 2 h. The flexural strength of the three test blocks was tested separately, and the final average value was the 2-h flexural strength of the test blocks. The specimens broken after the flexural test were maintained for 3d and tested for 3d compressive strength after baking to constant weight in an oven at (40 ± 4) °C. The compressive strength was determined by a microcomputer-controlled electronic pressure tester (305F-2, Shenzhen WANCE Test Equipment Co., Ltd., China) with a loading area of 0.0016 m^2^. The flexural strength was determined by an electric flexural tester (DKZ-6000, Wuxi JIANYI Instrument & Machinery Co., Ltd., China). α-HH strength standard was measured with reference standard of the building materials industry of the People's Republic of China (JC/T 2038–2010)^38^.

## Result and discussions

### Flotation test results

#### Frother screening

**Figure 1:**
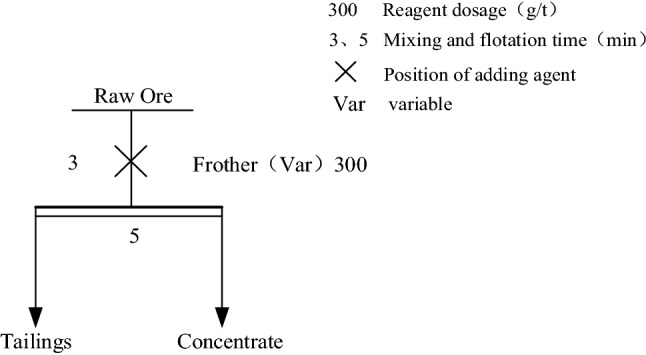
Flow chart of frother screening test.

The addition of a frother can remove the organic matter and fine slime in PG and remove part of the phosphorus enriched in the fine slime. Firstly, several commonly used frother were screened, The type of foaming agent used is chosen from Alcohols, ethers, and lipids are frequent ingredients in foaming agents such as Pine oil, MIBC, Tributyl phosphate (P86)^39–41^, the effects of Pine oil, MIBC, Tributyl phosphate (P86), on the flotation behavior of easy-to-float organic impurities in PG were compared. The test procedure is shown in Fig. 1, and the dosage of the agents is tentatively set at 300 g/t. The test results are shown in Table 1.

**Table 1:** Frother type screening test results.

Type	Concentrate			Tailings		
	Productivity/%	CaSO_4_·2H_2_O purity/%	Whiteness/%	Productivity/%	CaSO_4_·2H_2_O purity/%	Whiteness/%
Pine oil	94.8	90.26	23.2	5.2	60.93	11.6
MIBC	94.9	92.54	27.7	5.1	61.42	11.4
Tributyl phosphate (P86)	95.8	89.71	22.7	4.2	57.73	8.9

From the results of the screening test, it can be seen that several agents can flotation remove part of the easily floatable organic impurities. However, after comparison, the PG concentrate obtained by MIBC reverse flotation reached 27.7% whiteness and 92.54% purity. Comparing the productivity, whiteness, purity and other indexes, MIBC has the best effect in removing impurities. In addition, during the experiment, it was found that MIBC had a moderate foaming rate and foam structure compared with other foaming agents, mainly due to the strong foaming ability of MIBC, no trapping effect on gypsum minerals, and less influence of pH value, so MIBC was used as a foaming agent for removing floatable organic impurities and microfine mineral sludge in PG in the subsequent experiment. And from the comparison of the data obtained from several other flotation agents in Table 1, it is concluded that the whiteness of PG is positively related to the purity, and the higher the purity, the higher the whiteness.

#### Frother usage

After the frother screening test, MIBC was determined as the optimal frother for the removal of floatable organic impurities and slime, and the subsequent MIBC dosage test was conducted. The results are shown in Table 2. It can be seen that with the increase of MIBC dosage, the removal rate of organic impurities and slime, the whiteness and purity of PG concentrate increased. When the dosage of MIBC was 300 g/t, the PG concentrate index reached the best, and the PG flotation index was decreased gradually when the dosage of MIBC continued to increase, Mainly due to the excessive foaming agent will form a large number of sticky and fine bubbles, easy to bring out the part of the phosphogypsum adhering to the bubbles, reducing the productivity and grade of phosphogypsum concentrate. Therefore, in the follow-up test, the MIBC dosage was set at 300 g/t.

**Table 2:** Frother dosage test results.

Dosage/(g/t)	Concentrate			Tailings		
	Productivity/%	CaSO_4_·2H_2_O purity/%	Whiteness/%	Productivity/%	CaSO_4_·2H_2_O purity/%	Whiteness/%
50	92.5	91.21	26.2	7.5	62.36	12.9
150	91.4	90.18	25.9	8.6	65.91	14.7
250	90.8	91.30	26.2	9.2	65.48	14.5
300	94.9	92.54	27.7	5.1	61.42	11.4
350	85.7	91.74	26.7	14.3	67.83	15.2
400	83.9	91.86	26.3	16.1	70.10	16.3

#### Grinding test before desliming

**Figure 2:**
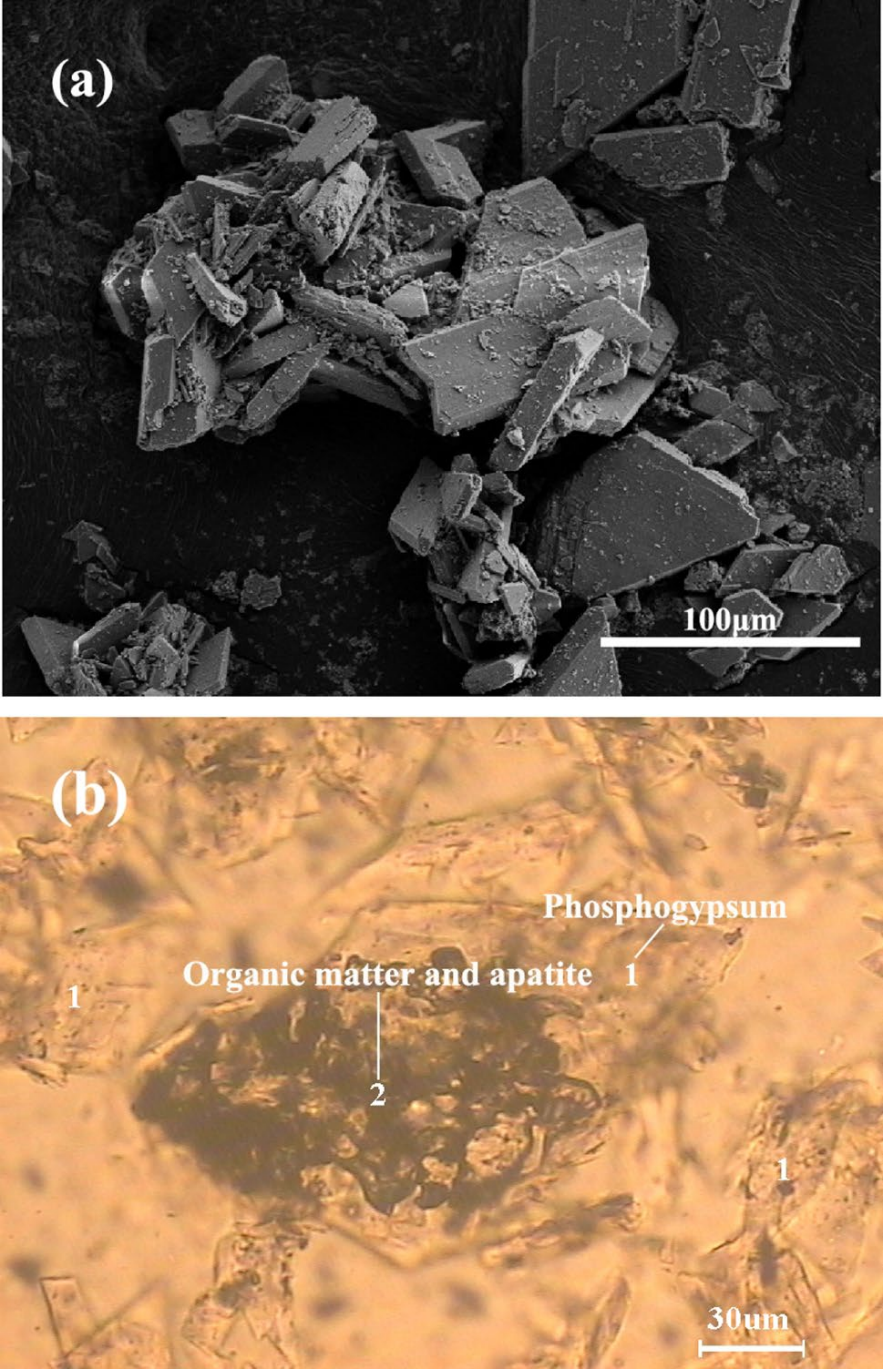
Reverse flotation desliming concentrate: (**a**) SEM, (**b**) polarizing microscope.

**Figure 3:**
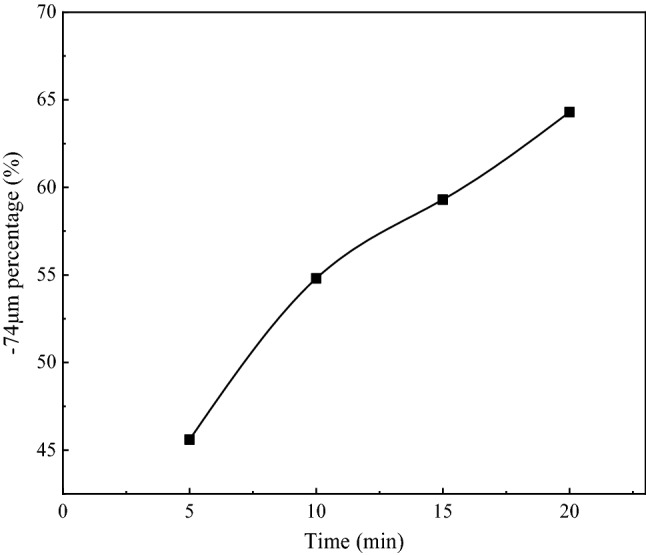
Proportion curve of grain-size after grinding.

**Figure 4:**
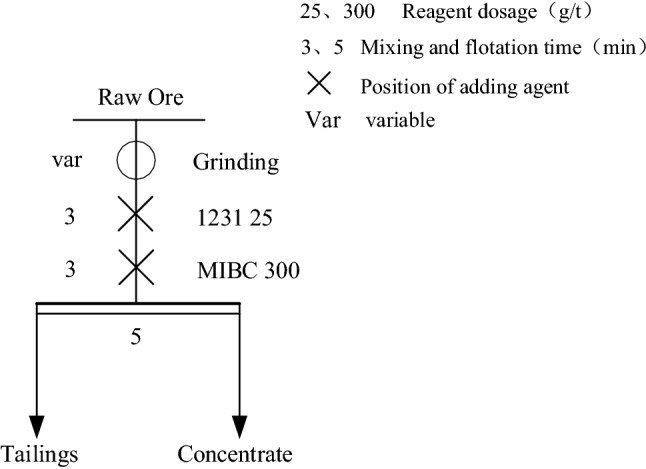
Flow chart of grinding test before desliming.

The results of polarized light microscopy and scanning electron microscopy analysis of MIBC reverse flotation desliming concentrate are shown in Fig. 2. According to the figure, it can be found that there are still a large number of flaky crystals in the PG concentrate aggregating with each other. Some of the gypsums are wrapped with black–brown organic matter and apatite, which has not been dissociated monomerically. A large number of aggregated crystals will affect the removal of organic matter. Therefore, in the flotation purification process should first be slightly grinding PG thus breaking up the aggregates, so that more surface impurities and gypsum to get separated. According to the preliminary exploration test on ball mill material ball ratio and water addition, the material to ball ratio of the ball mill was 3:1, and the water volume was 500 ml, and the effect on the flotation behavior of the floatable organic impurities in PG was observed by varying the grinding time. The grinding grain size distribution curve shown in Fig. 3. The test flow is shown in Fig. 4, and the test results are shown in Table 3.

**Table 3:** Grinding test results before desliming.

Time/min	− 0.074 mm percentage/%	Concentrate			Tailings		
		Productivity/%	CaSO_4_·2H_2_O purity/%	Whiteness/%	Productivity/%	CaSO_4_·2H_2_O purity/%	Whiteness/%
5	45.6	83.5	91.65	26.2	16.5	71.02	16.7
10	54.8	84.6	93.41	28.3	15.4	70.17	16.0
15	59.3	87.6	93.96	29.8	12.4	62.89	13.9
20	64.3	86.5	92.13	28.5	12.6	68.26	14.5

As can be seen from Table 3, the productivity, whiteness, and purity of PG concentrate showed an increasing trend with the increase of grinding time. As can be seen from Fig. 3, after 15 min grinding, the particle size content − 0.074 mm reaches 59.3%, the growth trend reached its peak, and the whiteness of PG concentrate obtained by reverse flotation reached 29.8%, and the purity reached 93.96%, which was the optimal condition for reverse flotation desliming, so the particle size content of samples − 0.074 mm was set at 59.3% in the subsequent test.

#### Dodecylamine collector dosage

**Figure 5:**
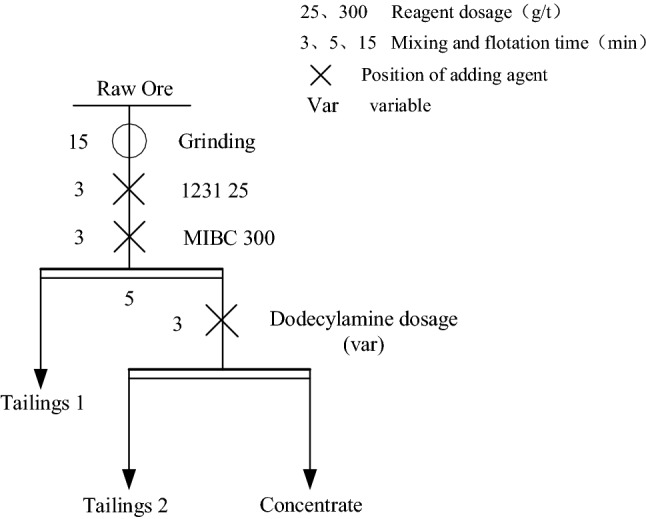
Flow chart of dodecylamine dosage test.

After the grinding and reverse flotation of PG to remove easy to float organic impurities and fine slime, it still contains some impurity minerals, mainly some coarse size of calcium phosphate stone. These impurities still affect the PG whiteness, and elemental phosphorus, etc. will be enriched in these impurities, so it is necessary to separate the gypsum from these residual impurities. Since the surface of CaSO_4_·2H_2_O is negatively charged in most of the pH range, the cationic collector has good collecting ability for it^42^. In this experiment, dodecylamine was selected for the separation and purification of PG, and the effect of dodecylamine dosage on the whiteness and purity of gypsum was investigated. The experimental procedure is shown in Fig. 5, and the results are shown in Table 4.

**Table 4:** Dodecylamine dosage test results.

Dosage/(g/t)	Product	Productivity/%	CaSO_4_·2H_2_O purity/%	Whiteness/%
25	Concentrate	28.6	93.58	28.5
	Tailings 1	18.1	70.26	16.9
	Tailings 2	53.3	91.39	28.6
50	Concentrate	49.1	93.96	29.8
	Tailings 1	19.1	71.99	17.8
	Tailings 2	31.8	91.82	29.2
100	Concentrate	71.6	93.67	28.9
	Tailings 1	15.7	65.29	15.9
	Tailings 2	12.7	88.74	22.9
150	Concentrate	78.9	94.16	34.5
	Tailings 1	15.4	70.92	17.2
	Tailings 2	5.7	72.37	19.7
200	Concentrate	76.3	93.85	29.9
	Tailings 1	16.8	68.29	16.6
	Tailings 2	6.9	73.36	18.3

From Table 4, it can be seen that the yield of PG concentrate increased with the increase of dodecylamine dosage. When the amount of dodecylamine reached 150 g/t, the continued increase of dosage led to the PG concentrate rate, whiteness and purity had a decreasing trend, which can be inferred that the increase of collector dosage led to the increase of CaSO_4_·2H_2_O up-floating in PG, which is easy to entrain more impurities in the up-floating process. Considering the cost of dodecylamine, the dosage of dodecylamine was selected as 150 g/t in the flotation test.

#### Comparison of PG properties before and after purification by flotation closed-circuit test

**Figure 6:**
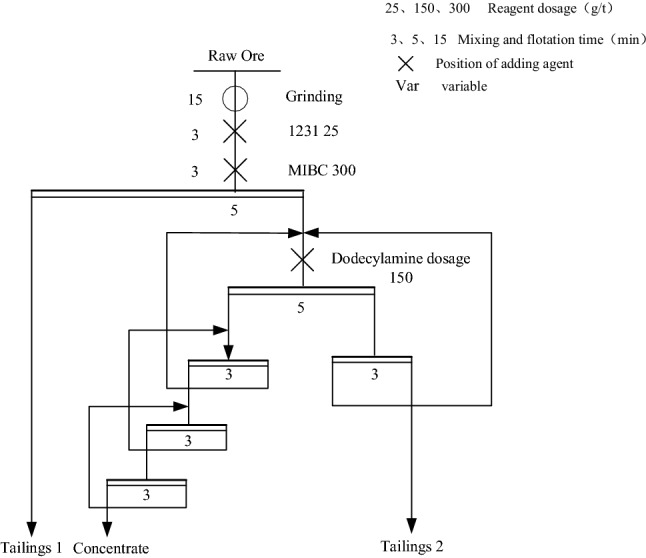
Flow chart of closed-circuit flotation test.

The flotation process of “one rough, one sweep and three fine” was adopted, which can get a better flotation index process. The PG closed-circuit flotation process is shown in Fig. 6, and the test results are shown in Table 5. Compared with the raw PG, the PG concentrate treated by the closed-circuit flotation condition has less impurity content and therefore has a higher utilization value. The total phosphorus content of the purified PG concentrate was 1.17%, and the soluble phosphorus content was reduced from 0.48 to 0.07%. Most of the soluble phosphorus and fluorine went into the beneficiation tailing water. The whiteness of PG was increased from 19.4 to 40.5%, and the purity was increased from 73.12 to 94.37%. The purified PG concentrate has met the national standard PG (GB/T 23456-2018) first-class product standard for gypsum building materials.

**Table 5:** Chemical composition, purity and whiteness of PG raw ore and PG concentrate (wt%).

	PG ore	PG concentrate	PG tailings 1	PG tailings 2
SO_3_	49.33	51.64	45.27	48.70
CaO	41.31	42.88	34.13	38.12
SiO_2_	5.03	2.76	11.93	4.51
P_2_O_5_	1.49	1.17	2.07	2.81
Al_2_O_3_	1.42	0.72	3.39	2.51
Fe_2_O_3_	0.58	0.30	1.77	0.89
SrO	0.35	0.20	0.42	1.63
K_2_O	0.19	0.09	0.32	0.17
TiO_2_	0.10	–	0.28	0.28
BaO	0.07	0.08	0.11	0.09
Na_2_O	0.07	–	0.04	0.03
Y_2_O_3_	0.02	0.02	0.01	0.02
ZnO	0.01	–	0.02	–
Whiteness	19.4	40.5	17.4	20.5
Purity	73.12	94.37	69.31	72.67

**Figure 7:**
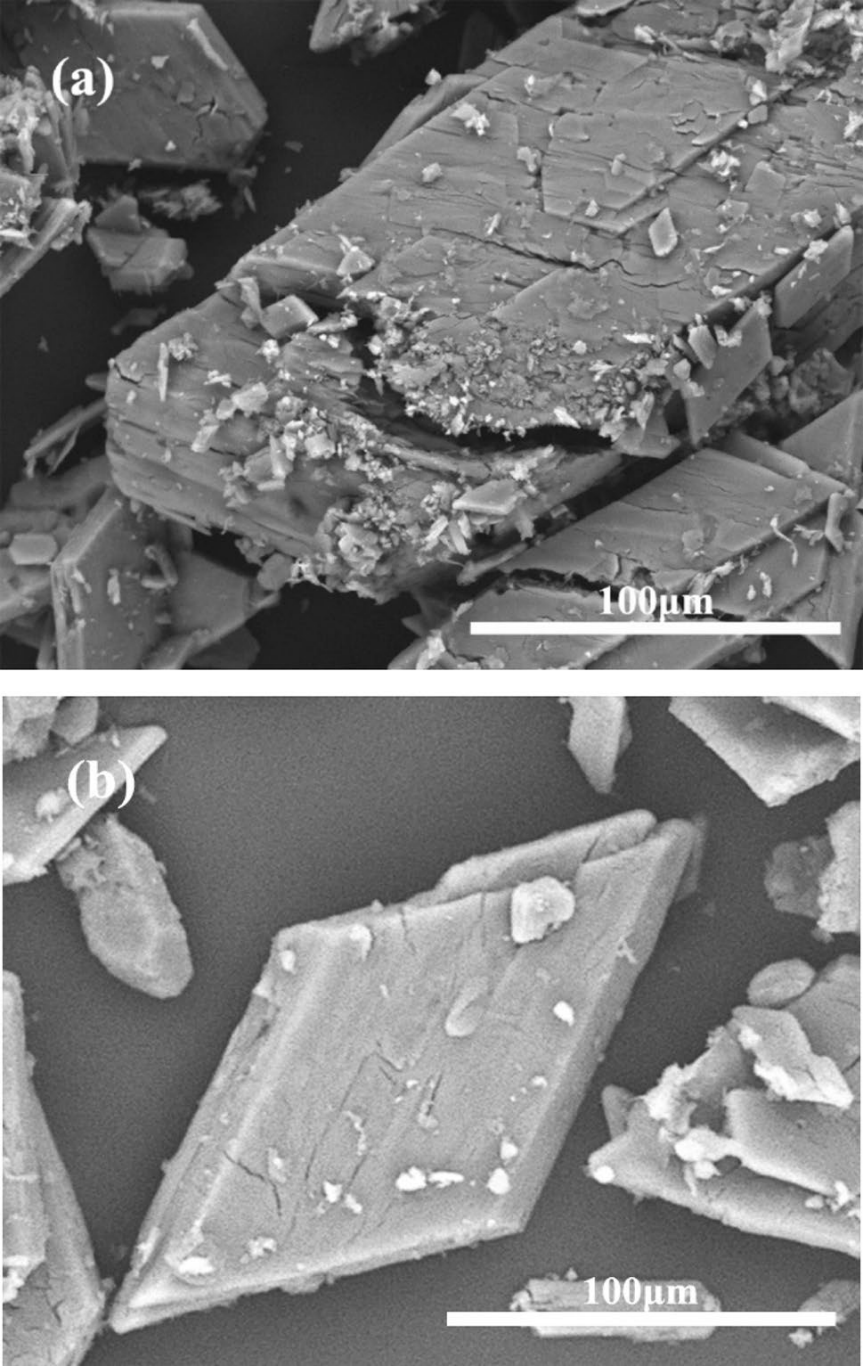
(**a**) SEM images of PG raw ore and (**b**) PG concentrate.

The SEM analysis of the concentrate and the original ore is shown in Fig. 7. Figure 7b indicates that the PG concentrate has a rhombic, plate-like structure, and the crystalline surface is flatter than the PG original ore, with significantly fewer surface impurities and no apparent defects. From Fig. 7a, it is seen that there are a large number of impurity particles on the surface of PG raw ore, and most of the plate structure is fractured and defective.

### Preparation and characterization of α-hemihydrate gypsum

**Figure 8:**
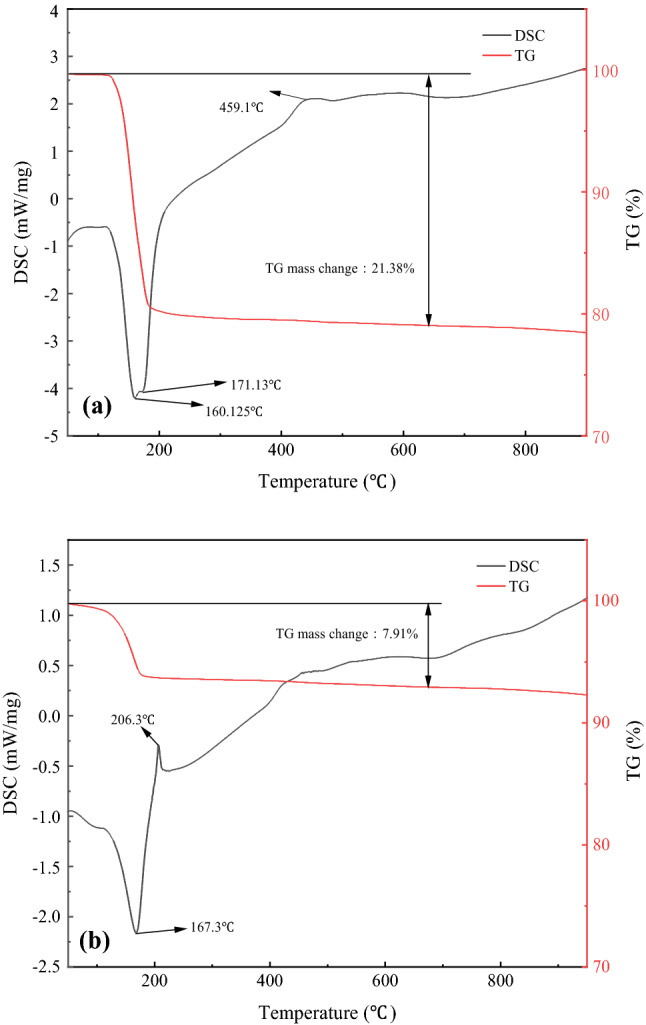
DSC/TG curves for (**a**) PG concentrate and (**b**) the converted product.

**Figure 9:**
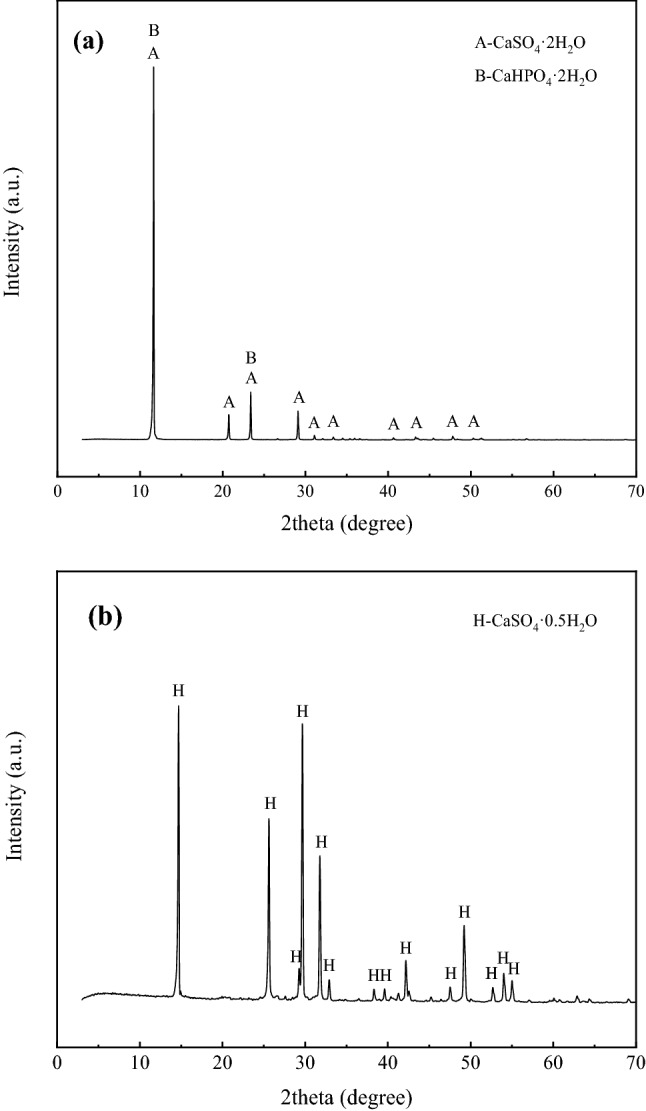
XRD for (**a**) PG concentrate and (**b**) the converted product.

Firstly, the preparation of α gypsum using PG concentrate without the addition of the crystal modifier was investigated. Figure 8 show the thermogravimetric analysis of α gypsum prepared from PG concentrate and α-hemihydrate gypsum, respectively. From Fig. 8a, it can be seen that the TG mass change for the PG concentrate is 21.38%, and the TG mass change for the α-HH is 7.91%, indicated that the two water of crystallization of CaSO_4_·2H_2_O crystal in the concentrate has been reduced to 0.5. The DSC curves showed heat absorption peaks at 160.125 °C and 171.13 °C, indicating that the water in PG crystals changed from 2 molecules to 1.5 molecules and then to 0.5 molecules^43^. An obscure exothermic peak was detected at 459.1 °C, indicating that the hard gypsum underwent a crystalline transformation. The results of the thermal analysis can verify that the main phase of PG is CaSO_4_·2H_2_O. From Fig. 8b, we can see that the heat absorption peak appears around 167.3 °C, followed by the exothermic peak around 206.3 °C, which is the unique characteristic peak of α-HH^44–46^, and no characteristic peak of CaSO_4_·2H_2_O phase was detected by XRD analysis (Fig. 9). The results verified that the CaSO_4_·2H_2_O crystals had been completely transformed into α-HH crystals.

**Figure 10:**
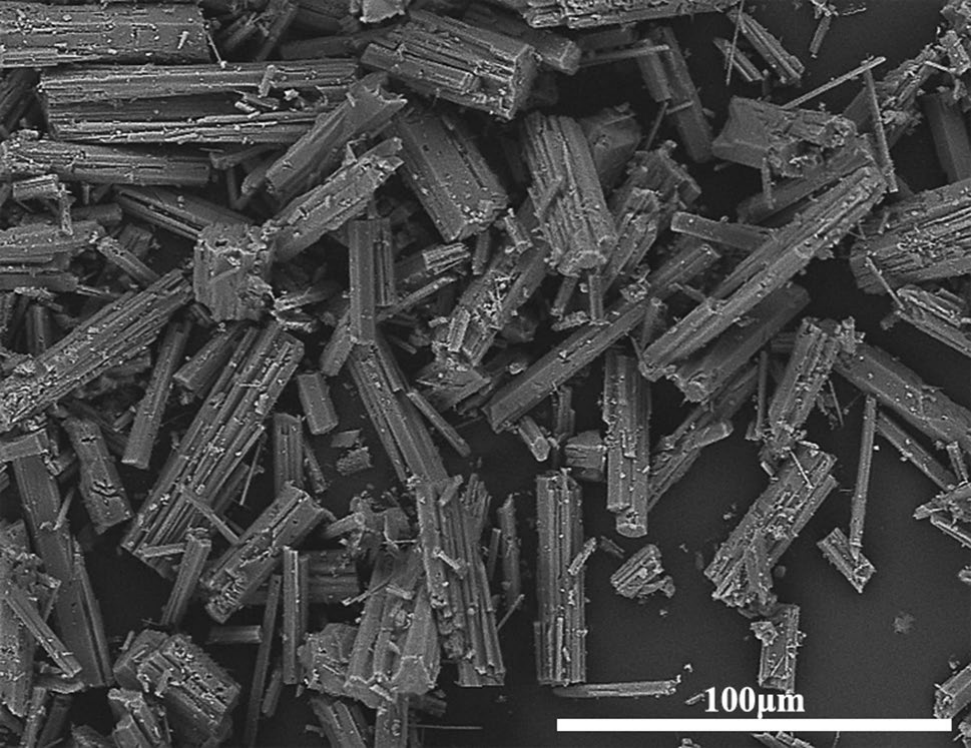
SEM image of unmodified converted crystal.

Figure 10 shows the SEM images of α-HH crystals transformed without the addition of crystal modifier, as shown in the figure, the length of the prepared α-HH crystals was in the range of 54–98 μm (average value of 76 μm), and the diameter was 5–10 μm (average value of 8 μm). The morphology of the transformed α-HH crystals was irregular hexagonal prisms. Since α-HH has various crystalline forms (plate, rod, column, etc.), and the different crystalline forms lead to different mechanical strengths. Usually, the short column α-HH with a complete crystalline form has higher mechanical strength^47,48^. However, α-HH will only form long columnar and needle-like crystals without the modifier, resulting in its low strength. Therefore, the unmodified α-HH crystals should be adjusted to smaller length and diameter crystals to improve their strength and performance.

### α-HH crystal shape modulation

**Figure 11:**
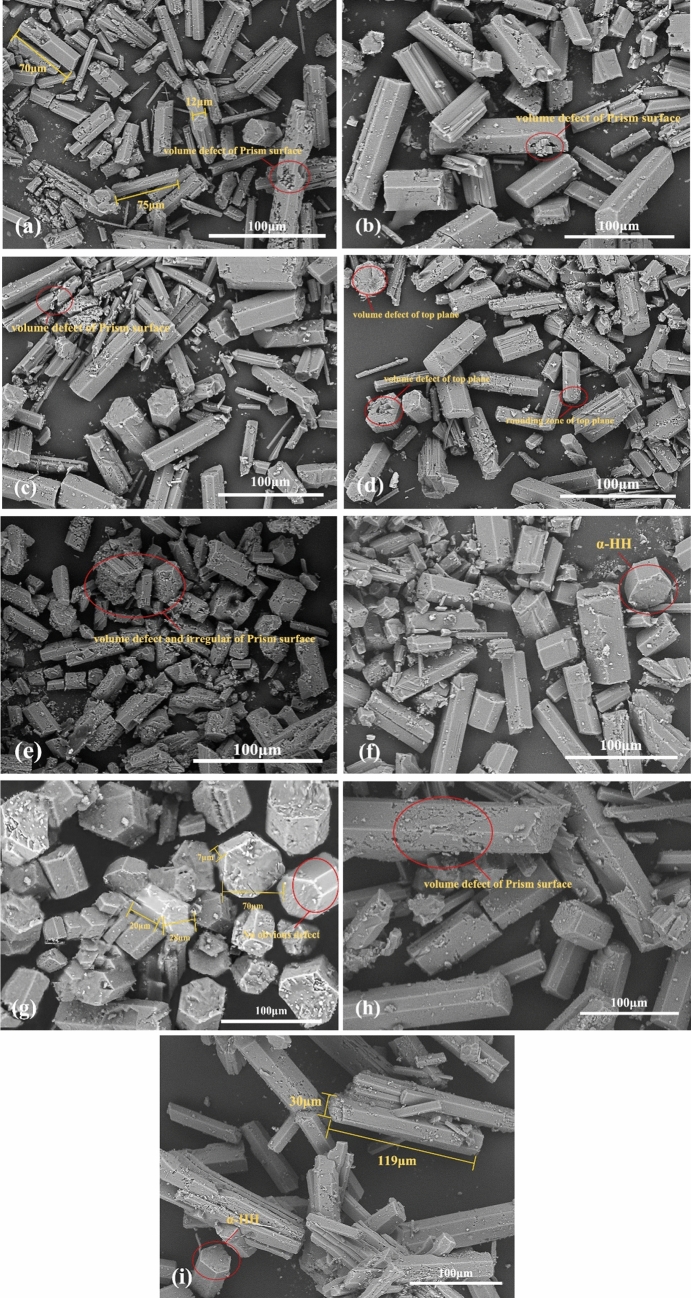
SEM images of the converted crystals with different dosage of maleic acid. The dosage: (**a**) = 0.01 wt%, (**b**) = 0.03 wt%, (**c**) = 0.05 wt%, (**d**) = 0.07 wt%, (**e**) = 0.09 wt%, (**f**) = 0.11 wt%, (**g**) = 0.13 wt%, (**h**) = 0.15 wt%, (**i**) = 0.17 wt%.

The other experimental conditions for the preparation of α-HH were kept consistent, by the preliminary study and literature search, the mixed solution of maleic acid and aluminum sulfate was selected as the crystal transfer agent, and the amount of fixed aluminum sulfate was 0.2%^49^. The amount of aluminium sulfate was fixed at 0.2 wt% of the sample mass. The α-HH crystals were adjusted by adding different amounts of Maleic acid (0.01 wt%, 0.03 wt%, 0.05 wt%, 0.07 wt%, 0.09 wt%, 0.11 wt%, 0.13 wt%, 0.15 wt%, 0.17 wt%). The effect of Maleic acid dosage on the morphology of PG crystals was analyzed using SEM, and the results are shown in Fig. 11. As seen from the figure, when the amount of Maleic acid was increased from 0.01 to 0.13 wt% (Fig. 11a–g), the length of α-HH crystals became shorter, the diameter increased, and the L/D ratio decreased. The crystal morphology gradually changed from long columnar to shortly columnar, and finally, the L/D ratio was reduced from 6.3 to 0.7 at the dosage of 0.13 wt%. When the dosage of Maleic acid exceeded 0.13 wt% (Fig. 11h–i), the α-crystal length gradually became longer and smaller in diameter, and the L/D ratio increased from 0.7 to 3.9.

The change of crystal morphology is determined by the relative growth rates of different crystalline planes, so the addition of crystal modifier to the reaction system can change the external shape of the crystal^45^. This theory is confirmed by the different morphologies of α-HH crystals shown in Fig. 11, where the change in crystal morphology can be interpreted as the adsorption of modifier on a specific crystal face, thus changing the relative growth rate of that face. Since the Ca^2+^ content of the top surface of α-HH crystals is higher than that of the prismatic surface, the crystal surface parallel to the c-axis is usually positively charged and has higher surface energy, and has a higher relative growth rate during the growth process^46,50^. When Maleic acid was added, the hydroxyl group adsorbed with Ca^2+^ on the top surface and formed a complex, which inhibited the growth of crystalline surfaces along the c-axis and coordinated the growth rates of different crystalline surfaces, and finally obtained α-HH crystals with relatively small L/D.

When the amount of Maleic acid was from 0.01 to 0.09 wt%, the top surface of some α-HH crystals was defective. When the amount of Maleic acid was from 0.11 to 0.17 wt%, the top surface of the crystals was basically flat, which indicated that the modifier mainly affected the growth of the top surface of α-HH crystals. Further observation of the defective part of the top of the crystal shows that most of the defective area is the middle area of the top surface. Therefore, it can be assumed that when the Maleic acid concentration is relatively low (0.01–0.09 wt%), which belongs to the initial stage of crystal growth, the modifier partially adsorbs on the top surface of the crystal, inhibiting the growth of the crystal along the c-axis direction and accelerating the growth of the crystal diameter, resulting in a radial expansion of the top surface. With the consumption of the modifier, the protruding part of the top surface of the crystal could not be adsorbed by the modifier, and the growth rate of these areas not adsorbed to the modifier was gradually larger than the central area, which eventually led to the defect of the top surface of the crystal. However, when the concentration of Maleic acid is relatively high (0.11–0.17 wt%), the amount of modifier is sufficient to adsorb on the whole top surface of the crystal, so there is no obvious defect on the surface.

**Figure 12:**
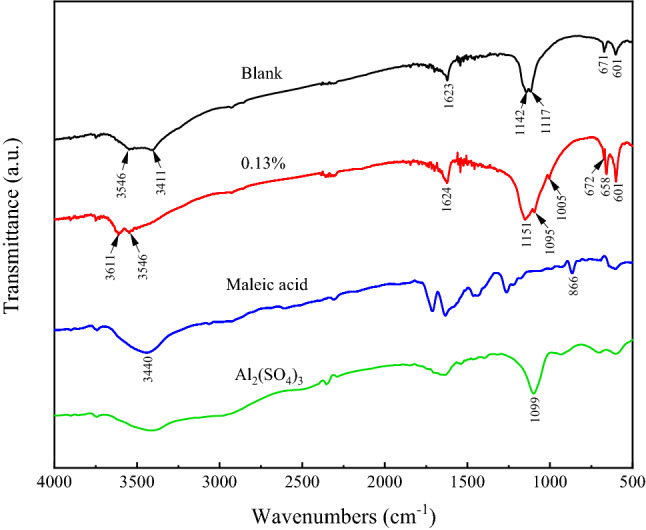
FTIR analysis results of modified sample and unmodified sample.

After analysis by SEM image, it was found that the crystal morphology of α-HH had reached the best state when the amount of Maleic acid was 0.13 wt% (Fig. 11g), so the α-HH was prepared with the amount of crystal modifier of 0.13 wt% was chosen. The results of the modified sample compared with the unmodified sample by FTIR analysis are shown in Fig. 12. The modified sample showed new absorption peaks at 1005 cm^−1^ and 672 cm^−1^, while the characteristic peak of the C–H group existed at 866 cm^−1^ for Maleic acid, indicating that Maleic acid interacted with the crystal to fix the C–H group on the crystal. And the new absorption peak at 1005 cm^−1^ may be due to the stretching vibration peak of SO_4_^2−^ of Aluminium sulfate at 1099 cm^−1^. Further observation of the spectra of the modified samples revealed that the peaks at 3611 cm^−1^ and 3546 cm^−1^ were shifted to the left by 65 cm^−1^ and 135 cm^−1^, respectively, compared with the wave numbers of the unmodified sample. It can be inferred that the carboxylate anion in Maleic acid located at 3440 cm^−1^ formed a complex with Ca^2+^ on the top surface of the crystal, which further verified the interaction between the modifier and the α-HH crystal.

### Comparison of the performance of PG raw ore and concentrate preparation of α-HH

The α-HH was prepared using PG raw ore and concentrate under the same conditions to compare the differences in the properties of the α-HH prepared from the two raw materials. The results of the study are shown in Table 6. The whiteness of α-HH prepared from PG raw ore and concentrate was increased from 27.8 to 46.3. The initial setting time and final setting time of the concentrate were extended by 3 min 10 s and 7 min 40 s respectively, compared with the original ore. It is due to the enhanced purity of the purified phosphogypsum, which converted into more hemihydrate gypsum than the original ore under the same circunstances, resulting in more water requirement and a longer condensation time. 2 h flexural and compressive strength of purified PG was increased by 46.15% and 79.46% compared with the original ore, and 3d dry compressive strength was increased by 39.6% compared with the original ore. The strength of the prepared α-HH reaches the standard α40 grade of the building materials industry of the People's Republic of China^38^.

**Table 6:** Comparison of α-HH mechanical strength of PG raw ore and PG concentrate.

	α-HH (raw ore)	α-HH (concentrate)
Average L/D ratio	7.2	0.7
W/H ratio/%	68	70
Initial setting time/(min:s)	6:50	10:00
Final setting time/(min:s)	10:20	18:00
2 h flexural strength (MPa)	4.16	6.09
2 h compressive strength (MPa)	12.1695	21.8395
3d compressive strength (MPa)	29.3424	40.9614

**Figure 13:**
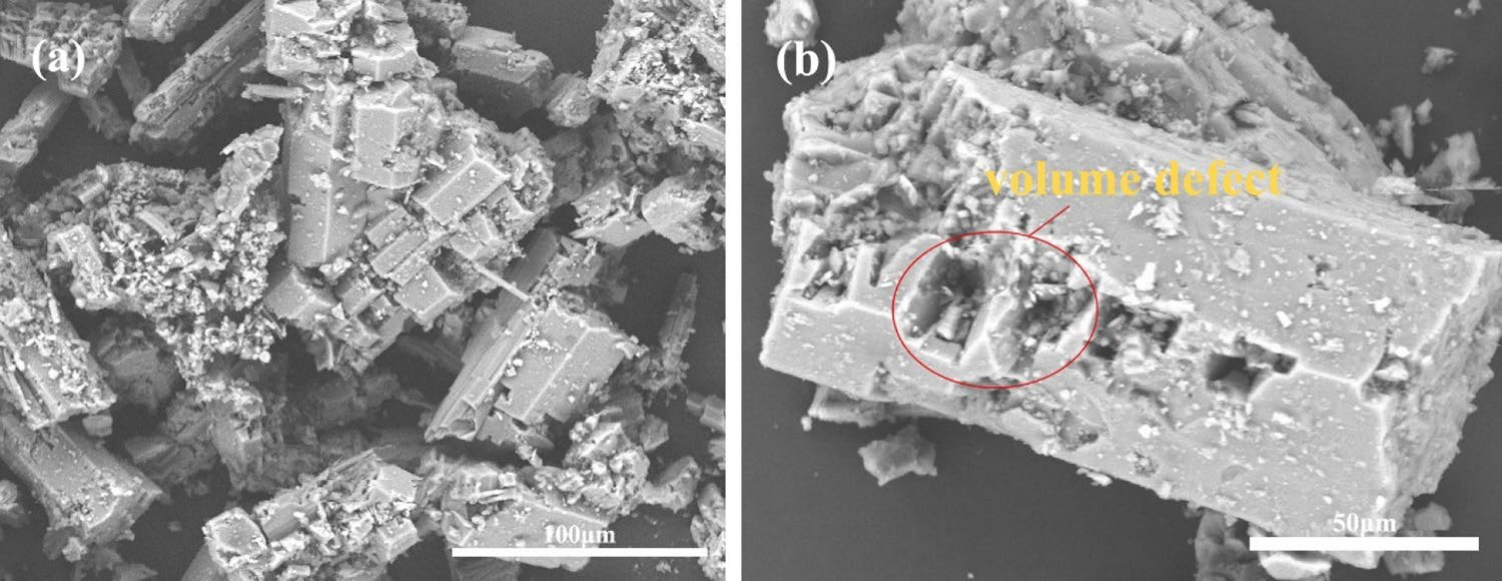
SEM images of converted crystals from PG raw ore (**a**) 100 μm, (**b**) 50 μm.

Comparative analysis of the SEM images of the crystals of the two products. Compared with Fig. 11g, the α-HH crystals prepared from PG raw ore (Fig. 13a) are smaller in size, longer in length and diameter, heavily agglomerated, and with more impurities on the surface. It can be assumed that some impurities in the PG raw ore accelerate the crystal transformation in the hydrothermal reaction, thus limiting the crystal size, and the smaller size crystals are more likely to agglomerate with each other. And some of the α-HH crystals prepared from PG raw ore showed fractures or defects on the surface (Fig. 13b), which may be due to the presence of impurities such as soluble phosphate on the surface of raw ore crystals, resulting in the deterioration of crystal morphology during the reaction^51^.

**Figure 14:**
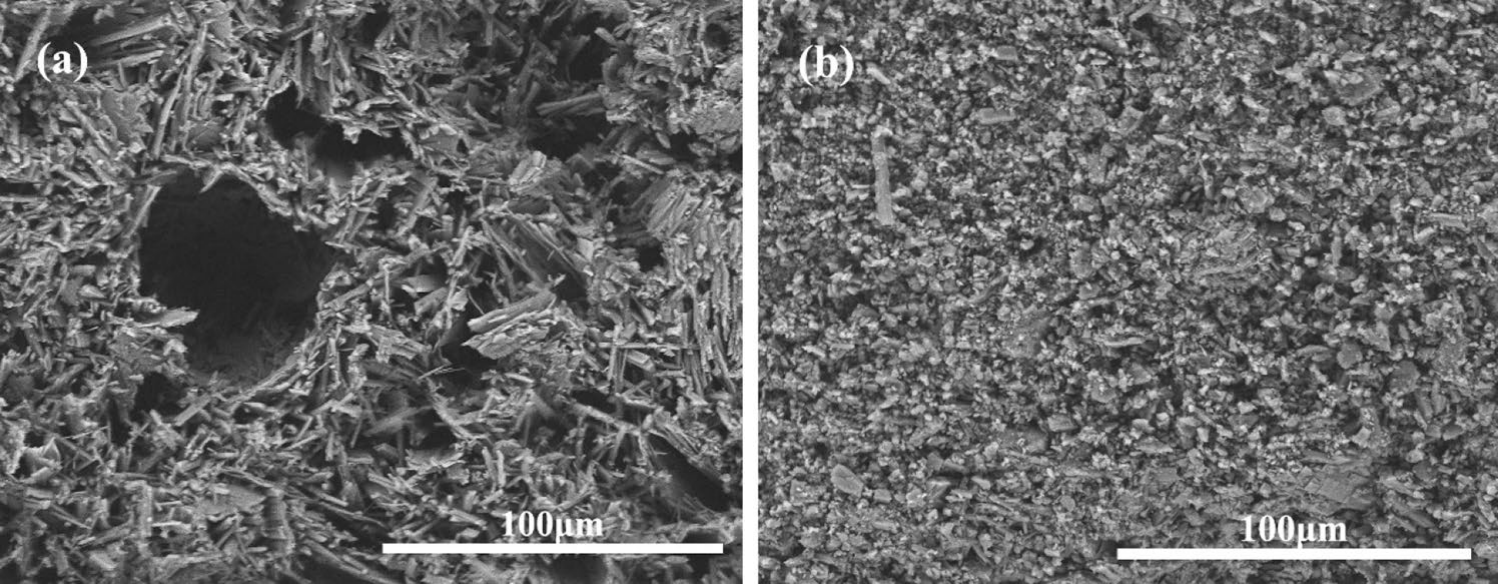
SEM image of sectional dense structure for (**a**) preparation of α-HH from PG raw ore and (**b**) preparation of α-HH from PG concentrate.

**Figure 15:**
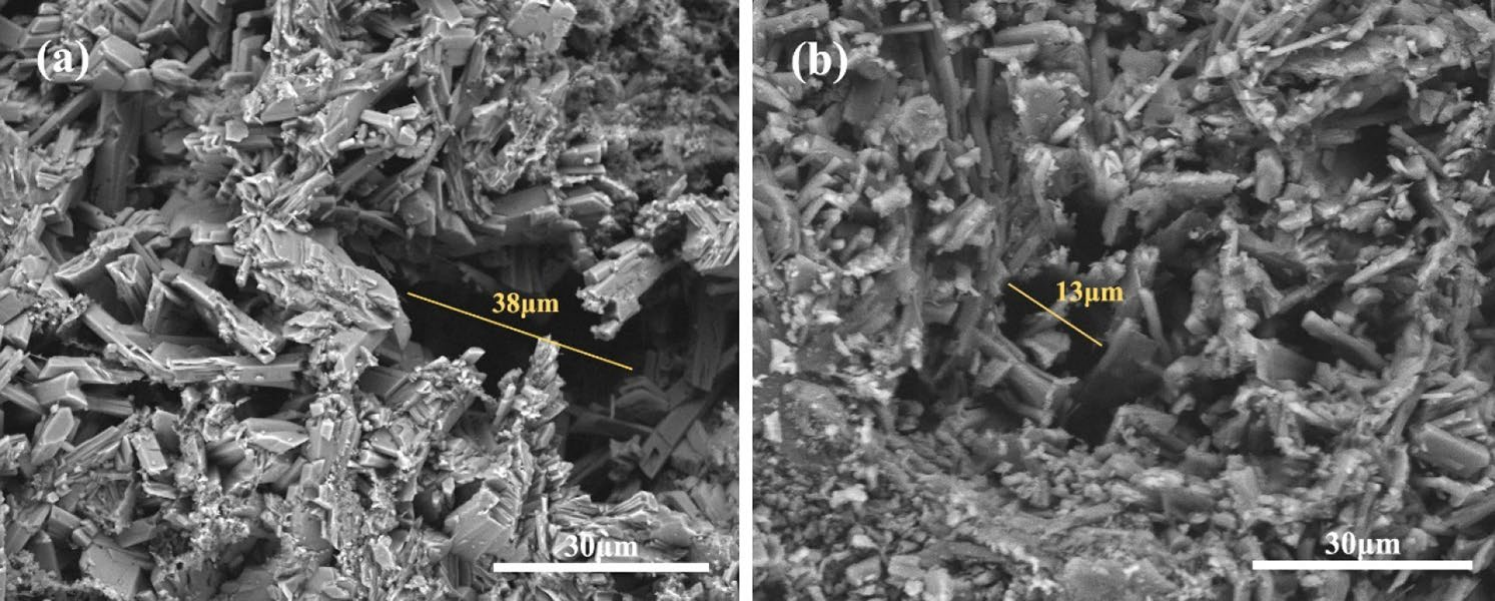
SEM image of aperture comparison for (**a**) preparation of α-HH from PG raw ore and (**b**) preparation of α-HH from PG concentrate.

The two α-HH products were prepared as specimens of the same size, and the differences in the fracture surfaces of the specimens were further observed (Fig. 14). It is apparent that the fracture surfaces of the α-HH specimens prepared from PG concentrate are relatively dense, while the fracture surfaces of the α-HH specimens prepared from PG raw ore are loose and have a large number of voids. From the comparison of the fracture surface cavity size of α-HH specimens prepared from two different raw materials in Fig. 15, it can be seen that the pore size in α-HH specimens purified by flotation shrinks from about 38 μm to about 13 μm. This is the fundamental reason for the improvement of the mechanical strength of α-HH specimens.

### Future research prospects

Follow-up experiments need to study the effective removal of soluble phosphorus and soluble fluorine from the test wastewater without affecting the rate of phosphogypsum concentrate. Use phosphogypsum concentrate to prepare other kinds of materials, broaden the application field of phosphogypsum concentrate and increase the added value of gypsum. Study the comprehensive utilization of two kinds of tailings produced by flotation so that the whole treatment process has zero waste discharge.

## Conclusion

In this study, the PG raw ore was purified using the flotation method, and then α-HH with different crystal shapes was prepared by hydrothermal reaction. The differences between the α-HH products prepared from PG raw ore and PG concentrate were discussed, and the following conclusions were obtained:

1. Adding frother MIBC can effectively remove organic matter and floatable slime from PG and then use dodecylamine to carry out flotation on PG. After treatment, the whiteness of PG concentrate reaches 40.5%, the purity of CaSO_4_·2H_2_O reaches 94.37%, and the soluble phosphorus content is reduced to 0.07%, which meets the national standard (GB/T 23456-2018) first-class product standard for gypsum building materials. It shows that the raw phosphogypsum has been effectively purified and refined. This process has lower energy consumption and cost than the previous process of purifying phosphogypsum, no secondary pollution, and is operable and can be widely used in industry subsequently.
2. As the amount of Maleic acid increased from 0 to 0.17 wt%, the L/D ratio of α-HH crystals decreased from 7.2 to 0.7. When the amount of Maleic acid was 0.13 wt%, the transformed α-HH products showed the best mechanical strength, the 2 h compressive and flexural strengths reached 21.8395 MPa and 6.09 MPa, respectively, and the 3d dry compressive strength was 40.9614 MPa. It has reached the national building material standard (α40 grade) and has the prospect of wide application.
3. Compared with the raw PG ore, the whiteness of α-HH product prepared from PG concentrate increased from 27.8 to 46.3. 2 h flexural and compressive strength increased by 46.15% and 79.46% compared with the raw ore, and 3d dry compressive strength increased by 39.6% compared with the raw ore.
